# Effect of Educational Activities on Pediatricians Prescribing Oral Antibiotics in Pediatric Emergency Rooms

**DOI:** 10.7759/cureus.98218

**Published:** 2025-12-01

**Authors:** Mie Mochizuki, Masanori Ohta, Makoto Nakamura, Toshihide Ishihara, Kiyomu Iijima, Daisuke Watanabe, Kazuya Takahashi, Hideaki Yagasaki, Isao Miyairi, Takeshi Inukai

**Affiliations:** 1 Department of Pediatrics, University of Yamanashi, Chuo, JPN; 2 Department of Pediatrics, Japan Pediatric Society in Yamanashi Prefecture, Yamanashi, JPN; 3 Department of Pediatrics, Hamamatsu University School of Medicine, Hamamatsu, JPN

**Keywords:** anti-bacterial agent, antimicrobial stewardship, drug prescription, emergency medical services, pediatrician

## Abstract

Background: Analyses of global antibiotic prescribing trends have identified Japan as one of the countries with the highest rates of antibiotic overprescription in pediatric outpatient care, thus indicating the necessity of antimicrobial stewardship for pediatricians.

Methods: We conducted two educational workshops for pediatricians in October 2018 and October 2019 in Yamanashi Prefecture, Japan, and then surveyed their effect on preferences for antibiotic prescription rates using questionnaires. Antibiotic prescribing in two pediatric emergency rooms for three years was also evaluated through health insurance claims. The primary objective is to evaluate changes in prescribing preferences, and the secondary objective is to assess changes in monthly prescribing rates and antibiotic class-specific prescribing rates.

Results: Immediately before the first workshop, 39.4% (28/71) of respondents agreed with prescribing antibiotics for a hypothetical case of mild acute otitis media. This percentage decreased to 16.4% (9/55) in the post-workshop survey. However, one year later, just before the second workshop, 44.0% (22/50) of respondents agreed with prescribing antibiotics. The oral antibiotic prescribing rate in pediatric emergency rooms decreased from 18.3% (4836/26,484 patient visits) in the baseline year (2017) to 12.4% (2868/23,188) and 12.2% (2651/21,709) in 2018 and 2019, respectively. The prescribing rate of cephems among all prescribed antibiotics decreased from 53.8% to 46.4% and 43.4%, while that of semisynthetic penicillin increased from 26.7% to 34.7% and 35.1%, respectively. A transient reduction in seasonal prescribing rate was observed in the autumn of 2018 and 2019, aligning with the timing of the workshops.

Conclusions: Antibiotic prescribing preferences and behavior among pediatricians changed following the workshops. However, the impact of these workshops gradually diminished over time, underscoring the importance of sustained antimicrobial stewardship interventions.

## Introduction

Antimicrobial resistance is a growing global problem threatening modern medicine. The World Health Organization (WHO) is aware of this crisis and adopted a global action plan on antimicrobial resistance in 2015. Subsequently, the Japanese Government launched the National Action Plan on Antimicrobial Resistance in 2016 [1].

In Japan, most antibiotics are administered orally in outpatient settings [2]. Notably, a global study based on wholesale data in 2015 showed that Japan is one of the worst countries (and the worst among 36 high-income countries) for overprescribing antibiotics in pediatric outpatient settings [3]. In a previous nationwide study of outpatient oral antibiotic prescriptions using the national health claims database from 2013 to 2016, the prescribing rate in Yamanashi Prefecture, one of 47 prefectures in Japan, was almost the same as the national average [4]. This finding indicates that antimicrobial stewardship is required for pediatricians in Yamanashi Prefecture.

Therefore, in this study, we established several educational workshops for pediatricians in Yamanashi Prefecture and evaluated the effect of these workshops on pediatricians’ preferences for antibiotic prescribing using questionnaires. We also evaluated the antibiotic prescribing rate in two pediatric emergency rooms.

## Materials and methods

Study design

**Figure 1:**
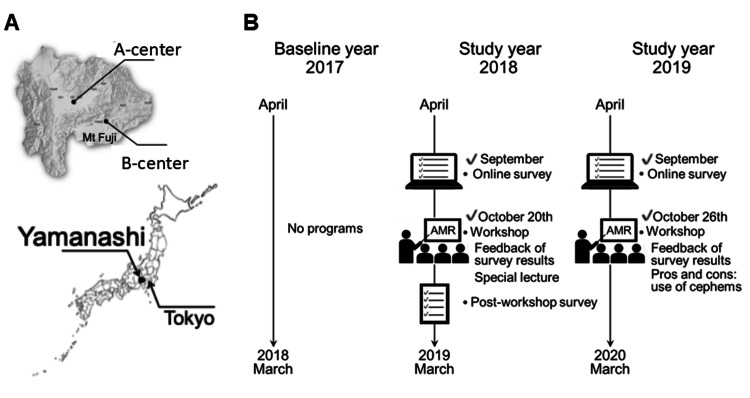
Design of this study (A) Location of Yamanashi Prefecture and the two emergency rooms. (B) Schematic representation of the study design.

This was an evaluation study of an intervention to decrease inappropriate antibiotic prescribing by pediatricians in pediatric emergency rooms in Yamanashi Prefecture, Japan. Yamanashi Prefecture comprises two medical regions separated by mountains (Figure 1, Panel A). In each medical region, a public emergency room is available for children younger than 15 years. Kofu and Fuji-Tobu pediatric emergency rooms are hereafter designated A-center and B-center in this study, respectively (Figure 1, Panel A).

Medical services are available in the A-center from 7:00 PM to 7:00 AM (opening hours: 12 hours) on weekdays, from 3:00 PM to 7:00 AM (16 hours) on Saturdays, and from 9:00 AM to 7:00 AM (22 hours) on Sundays and public holidays. Medical services are available in the B-center from 8:00 PM to 12:00 PM (four hours) weekdays, from 3:00 PM to 12:00 PM (nine hours) on Saturdays, and from 9:00 AM to 12:00 PM (15 hours) on Sundays and public holidays. Prescriptions are generally issued for one day at a time, except for Saturdays and public holidays.

In each emergency room, self-, hospital-, and university-employed pediatricians provide medical care in shifts. Seventy-two pediatricians (27 self-employed, 26 hospital-employed, and 19 university-employed) worked in the A-center, and 42 pediatricians (10 self-employed, 21 hospital-employed, and 11 university-employed) worked in the B-center. Twenty-five pediatricians who worked shifts in the B-center (four self-employed, 10 hospital-employed, and 11 university-employed) also worked shifts in the A-center, and the remaining 17 pediatricians (6 self-employed and 11 hospital-employed) covered approximately two-thirds of the shifts in the B-center.

The intervention included educational workshops, coupled with a questionnaire, to survey pediatricians’ preferences regarding antibiotics. The primary outcomes were changes in pediatricians’ antibiotic prescribing preferences and changes in antibiotic prescribing in two pediatric emergency rooms.

Questionnaire survey

The first online survey was carried out in September 2018 to determine a baseline of pediatricians’ antibiotic prescribing preferences (Appendix). The second online survey for comparison (using a questionnaire similar to the first with several identical questions) was carried out in September 2019. These questionnaires were sent to 141 members of the Japan Pediatric Society in Yamanashi Prefecture. Two online surveys were conducted using Google Forms. In addition, an onsite questionnaire, implemented on October 20, 2018, was provided to the participants of the first workshop, who also answered anonymously.

Intervention

An educational intervention workshop was prospectively conducted by core member pediatricians of the Japan Pediatric Society in Yamanashi Prefecture. In 2017 (the baseline year), no educational workshops were implemented (Figure 1, Panel B). In 2018 and 2019 (the study years), we conducted educational workshops on antimicrobial resistance with the aim of reducing inappropriate antibiotic prescribing in the outpatient clinic and emergency rooms. On October 20, 2018, we reported the results of the first online survey and presented a one-hour educational lecture entitled “Optimal antimicrobial medicines considering AMR in the pediatric outpatient clinic.” On October 26, 2019, we conducted another educational workshop, which included feedback on the results of the second online survey and a discussion on the advantages and disadvantages of using cephems.

Data collection and statistical analysis

Regarding overall antibiotic prescribing rates, we retrospectively reviewed the anonymized health insurance claim data of patients who visited two pediatric emergency rooms from April 2017 to March 2020. The study was conducted with the approval of the Ethics Committee of the University of Yamanashi (Approval No.: 2280). Retrospective reviews of health insurance claim data were performed in 2023 and 2024. Antibiotics were categorized into four groups: cephems, semisynthetic penicillins, macrolides, and others (e.g., fosfomycin, fluoroquinolones, and new quinolones). Prescriptions of antibiotic eye drops, ear drops, and ointments were not included in the analyses. Statistical analyses were conducted using Fisher’s exact test, and statistical significance was defined as p < 0.05.

## Results

Surveys on antibiotic prescribing preferences

For the baseline, no educational workshops were implemented in 2017. As shown in Figure 1, Panel B, two educational workshops were held in October 2018 and October 2019, during the study period. Three questionnaire surveys were administered: immediately before and after the first workshop and before the second workshop. Attendance at the first and second workshops included 76 members (53.9%) and 70 members (49.6%), respectively, out of a total of 141 members of the Japan Pediatric Society in Yamanashi Prefecture. A total of 71 members (50.4%) responded to the anonymous online survey conducted prior to the first workshop. The onsite post-workshop anonymous survey received responses from 55 members (39.0%), accounting for 72.4% of attendees. Similarly, 50 members (35.5%) completed the anonymous online survey conducted prior to the second workshop.

Among the three surveys, the most notable change in responses was observed for a question regarding a hypothetical case of mild acute otitis media (Table 1): “Do you prescribe antibiotics to an afebrile toddler who complains of mild otalgia with erythema of the eardrum?” A total of 28 out of 71 respondents (39.4%) answered “yes” in the first online survey, while nine out of 55 participants (16.4%) answered “yes” in the post-workshop survey. A significant decrease (p = 0.0057) from the baseline survey to the post-workshop survey was noted following a Fisher’s exact test. However, in the second online survey, 22 out of 50 respondents (44%) answered “yes”; hence, a significant increase (p = 0.0026) from the post-workshop survey to the second online survey was observed.

**Table 1:** Questionnaire surveys of antibiotic prescribing preferences The data are presented as the number of people who selected “Yes (prescribe antibiotics)” and their percentage. *p < 0.05 and **p < 0.05 indicate Fisher’s exact test between the first online survey and the post-workshop survey, and between the post-workshop survey and the second online survey, respectively.

		First online	Post-workshop	Second online
		Sep 2018	Oct 2018	Sep 2019
		N = 71	N = 55	N = 50
*Do you prescribe antibiotics in the following cases?*				
1	Afebrile toddler who complains of wet cough with clear post-nasal discharge	2 (2.8%)	2 (3.6%)	0 (0%)
2	Afebrile toddler who complains of wet cough with purulent post-nasal discharge	17 (23.9%)	10 (18.2%)	13 (26%)
3	Afebrile toddler who complains of mild otalgia with erythema in the eardrum	28 (39.4%)	9 (16.4%)*	22 (44%)**
4	Afebrile school-aged child who complains of wet cough without wheezing	6 (8.5%)	2 (3.6%)	3 (6%)

Although statistically insignificant, similar trends were observed in the question about a hypothetical case of mild sinusitis (Table 1): “Do you prescribe antibiotics to an afebrile toddler who complains of wet cough with purulent post-nasal discharge?” Specifically, 17 out of 71 (23.9%), 10 out of 55 (18.2%), and 13 out of 50 (26%) respondents answered “yes” in the first online survey, post-workshop survey, and second online survey, respectively. In the remaining 11 questionnaires, antibiotic prescribing preferences were largely similar among responders.

Changes in oral antibiotic prescribing

We next analyzed oral antibiotic prescriptions based on all available health insurance claims in the two centers (Table 2).

**Table 2:** Changes in antibiotic prescribing rates at two centers in Yamanashi Prefecture The data are presented as the number of prescriptions (prescription rate for the fiscal year).

	2017			2018			2019		
	A-center	B-center	Total	A-center	B-center	Total	A-center	B-center	Total
Patient visits	18,030	8454	26,484	15,612	7576	23,188	14,481	7228	21,709
Total prescriptions	3312	1524	4836	2009	859	2868	1874	777	2651
	(18.4%)	(18.0%)	(18.3%)	(12.9%)	(11.3%)	(12.4%)	(12.9%)	(10.7%)	(12.2%)
Semisynthetic penicillins	987	305	1292	741	255	996	683	247	930
	(5.47%)	(3.61%)	(4.88%)	(4.75%)	(3.37%)	(4.30%)	(4.72%)	(3.42%)	(4.28%)
Cephems	1642	961	2603	925	407	1,332	822	328	1150
	(9.11%)	(11.4%)	(9.83%)	(5.92%)	(5.37%)	(5.74%)	(5.68%)	(4.54%)	(5.30%)
Macrolides	632	250	882	319	191	510	344	181	525
	(3.51%)	(2.96%)	(3.33%)	(2.04%)	(2.52%)	(2.20%)	(2.38%)	(2.50%)	(2.42%)
Others	51	8	59	24	6	30	25	21	46
	(0.28%)	(0.09%)	(0.22%)	(0.15%)	(0.08%)	(0.13%)	(0.17%)	(0.29%)	(0.21%)

In the baseline year of 2017 (from April 2017 to March 2018), oral antibiotics were prescribed in 4836 out of 26,484 patient visits (18.3%) at both centers (3312/18,030 (18.4%) in the A-center and 1524/8454 (18.0%) in the B-center).

**Figure 2:**
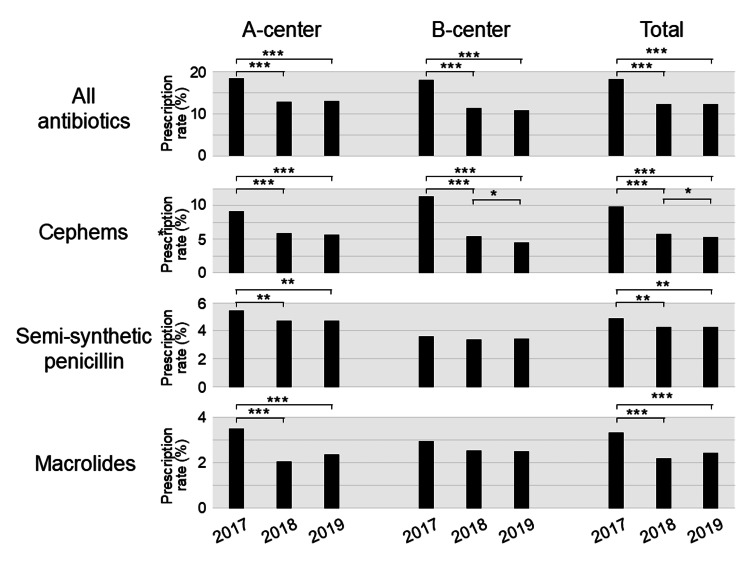
Changes in antibiotic prescribing rates Prescribing rates of all antibiotics, cephems, semisynthetic penicillins, and macrolides in A-center (left), B-center (middle), and in both centers (right). p-values of Fisher’s exact tests are indicated when statistically significant: *p < 0.05, **p < 0.01, and ***p < 0.001.

In the study year of 2018 (from April 2018 to March 2019), oral antibiotics were prescribed in 2868 out of 23,188 patient visits (12.4%) (2009/15,612 (12.9%) in the A-center and 859/7576 (11.3%) in the B-center), and the prescribing rate in 2018 significantly decreased by 32.3% overall (p < 0.0001), 30.0% (p < 0.0001) in the A-center, and 37.1% (p < 0.0001) in the B-center, compared with the 2017 baseline, respectively (Figure 2, Table 2).

In the study year of 2019 (from April 2019 to March 2020), oral antibiotics were prescribed in 2651 out of 21,709 patient visits (12.2%) (1874/14,481 (12.9%) in the A-center and 777/7228 (10.7%) in the B-center), and the prescribing rate in 2019 also decreased significantly by 33.1% overall (p < 0.0001), 29.6% (p < 0.0001) in the A-center, and 40.4% (p < 0.0001) in the B-center compared with the baseline in 2017, respectively (Figure 2, Table 2).

We next evaluated the types of prescribed oral antibiotics (Figure 2, Table 2). In the A-center, the prescribing rates of all three major antibiotic types in the study years of 2018 and 2019 significantly decreased from those in the baseline year of 2017. In the B-center, the prescribing rate of cephems in the study years significantly decreased compared to 2017. In total, in the two centers, prescribing rates of all three major antibiotic types in the study years (2018 and 2019) significantly decreased compared to the baseline year (2017).

**Figure 3:**
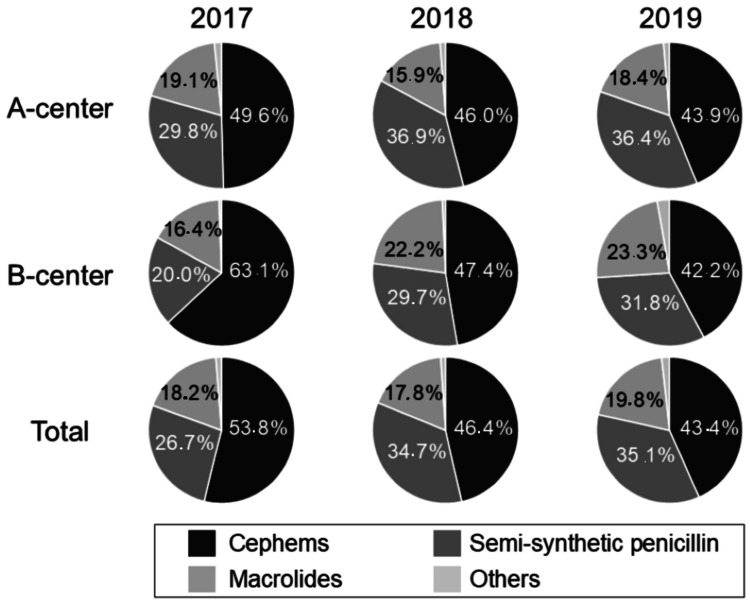
Changes in the types of prescribed oral antibiotics Percentages of antibiotic use of three major types (cephems, semisynthetic penicillins, macrolides, and others).

The most significant reduction in prescribing rate was seen in cephems (Figure 2, Table 2). In the baseline year of 2017, cephems were prescribed in 2603 out of 26,484 patient visits (9.8%) (1642/18,030 (9.1%) in the A-center and 961/8,454 (11.4%) in the B-center). Cephems accounted for 53.8% of the total prescribed antibiotics in two centers, while semisynthetic penicillin accounted for 26.7% (Figure 3). In the study year of 2018, cephems were prescribed in 1332 out of 23,188 patient visits (5.7%) (925/15,612 (5.9%) in the A-center and 407/7576 (5.4%) in the B-center), and the prescribing rate of cephems in 2018 significantly decreased by 41.6% overall (p < 0.0001), 34.9% (p < 0.0001) in the A-center, and 52.7% (p < 0.0001) in the B-center, respectively, compared with the rate in 2017. The prescribing rate of cephems among all prescribed antibiotics decreased to 46.4% in two centers, while that of semisynthetic penicillin increased to 34.7% (Figure 3).

In the study year of 2019, cephems were prescribed in 1150 out of 21,709 patient visits (5.3%) (822/14,481 (5.7%) in the A-center and 328/7228 (4.5%) in the B-center), and the prescribing rate of cephems in 2019 significantly decreased by 46.1% overall (p < 0.0001), 37.7% (p < 0.0001) in the A-center, and 60.1% (p < 0.0001) in the B-center, respectively. Moreover, the prescribing rate of cephems in 2019 was significantly decreased by 7.8% overall (p = 0.039) and 15.5% (p = 0.021) in the B-center compared with that in 2018, respectively. The prescribing rate of cephems decreased to 43.4% in two centers, while that of semisynthetic penicillin increased to 35.1% (Figure 3).

**Figure 4:**
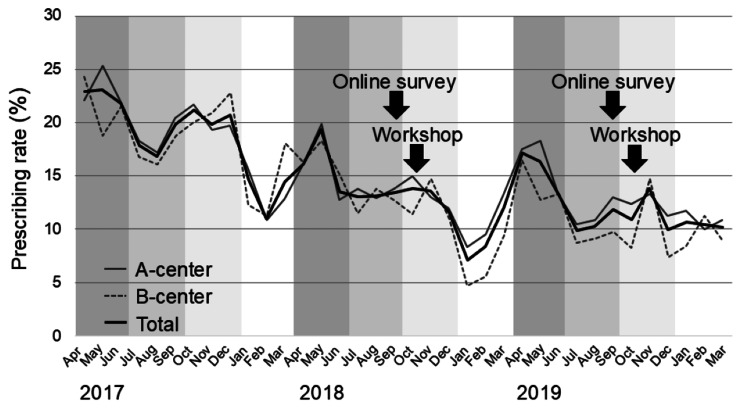
Changes in the monthly prescribing rate of oral antibiotics The gray, dotted gray, and bold black lines represent changes in the monthly prescribing rates of oral antibiotics at the A-center, B-center, and the combined total of both centers, respectively.

Although less significant in comparison with cephems, prescribing of semisynthetic penicillins and macrolides also decreased overall and in the A-center. We finally evaluated changes in the monthly prescribing rate (Figure 4).

In the baseline year of 2017, a seasonal change was observed; the prescribing rate was relatively higher in spring (April to June) and autumn (October to December) compared with that in summer (July to September) and winter (January to March).

**Figure 5:**
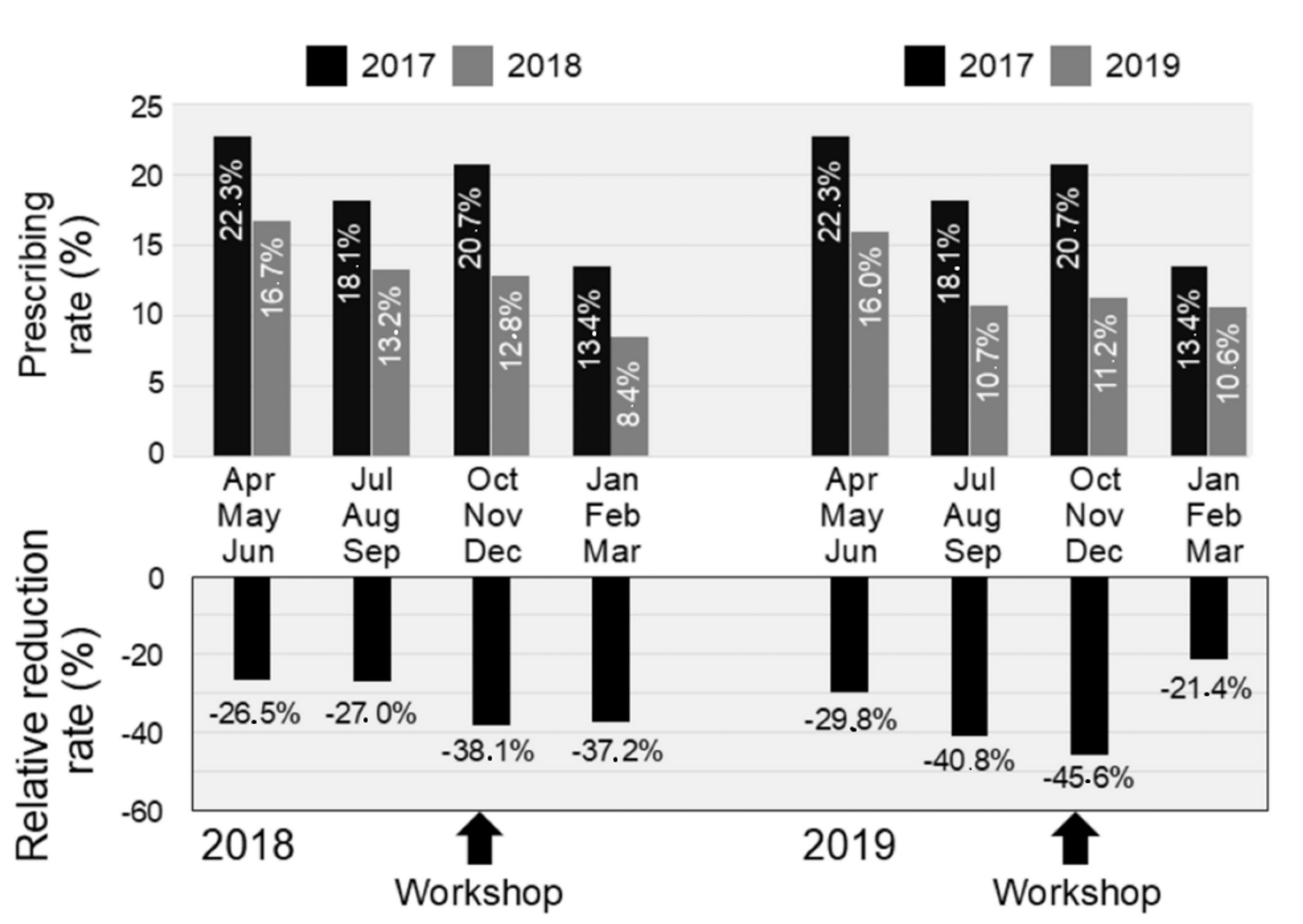
Changes in the seasonal prescribing rate of oral antibiotics In the top panel, the black and gray bars indicate prescribing rates for the baseline year (2017) and the study years (2018 and 2019), respectively. In the bottom panel, each bar indicates the relative reduction rate from the baseline year (2017) to the study years (2018 and 2019).

Thus, we also compared seasonal (three months) total prescribing rates (of two centers) in 2018 and 2019 with those in 2017 (Figure 5).

Before starting the stewardship, a decreased trend in prescribing rate was already seen in the spring (from 22.3% in 2017 to 16.7% in 2018, and the relative reduction rate was -26.5%) and summer (from 18.1% to 13.2%, and -27.0%) of 2018. The prescribing rate in autumn largely decreased from 20.7% in 2017 to 12.8% in 2018, and the relative reduction rate was -38.1%. The relative reduction rate remained at -37.2% (from 13.4% to 8.4%) in winter. A similar seasonal change was seen in 2019. The relative reduction rate in spring and summer was -29.8% (from 22.3% in 2017 to 16.0% in 2019) and -40.8% (from 18.1% to 10.7%), respectively. The relative reduction rate in autumn was -45.6% (from 20.7% to 11.2%), although it was -21.4% (from 13.4% to 10.6%) in winter.

## Discussion

Since the launch of Japan’s antimicrobial resistance action plan in 2016, various antimicrobial stewardship activities have begun [5]. In a nationwide study reported by Iwamoto et al. [6], nationwide activities resulted in antibiotic prescriptions for pediatric outpatients being decreased from 24.9 days of therapy per 1000 pediatric inhabitants/day in 2017 to 22.9 days of therapy per 1000 pediatric inhabitants/day in 2018 (8.0% reduction). In this study, the antibiotic prescribing rate in two pediatric emergency rooms decreased from 18.3% in 2017 to 12.4% in 2018, which was a 32.2% reduction. Therefore, although the reference index was not identical, the reduction observed in our two centers from 2017 to 2018 appears to be much higher than that observed in the above-mentioned nationwide study. This may be partly attributable to the impact of the first workshop conducted in the region.

In the present study, before starting our targeted activities, prescribing rates in spring and summer of 2018 were already reduced by 26.5% and 27.0% compared with those in spring and summer of 2017, respectively. These reductions appear to be greater than those found in the previous nationwide study [6]. In the beginning of 2018, although no specific activities had started, several voluntary members of the Japan Pediatric Society in Yamanashi Prefecture had already proceeded with preparations for educational workshops. Therefore, without starting specific workshops, such preparation activity in combination with the launch of the national action plan might have already affected the prescription behavior among some members of the Japan Pediatric Society in Yamanashi Prefecture.

Immediately after the first workshop in October 2018, we observed two main changes. First, there was a change in the answer to the questionnaires about antibiotic preference in the post-workshop survey. In the question about a hypothetical case of mild acute otitis media, a higher percentage of respondents agreed with antibiotic prescription in the first online survey before the first workshop than those who agreed in the post-workshop survey. Second, there was a change in the antibiotic prescribing rate in the two centers. In autumn 2018, the prescribing rate was markedly decreased by 38.1% compared with that in autumn 2017. These observations suggested that the first workshop affected the preference and prescribing of antibiotics, at least immediately after the workshop.

Although a large reduction in the prescribing rate was continuously observed in the winter of 2018 (-37.2%), the relative reduction rate in both centers recovered to -29.8% in the spring of 2019. Regarding the question about a hypothetical case of mild acute otitis media, 44.0% of respondents consistently agreed with antibiotic prescriptions in the second online survey performed in September 2019, which indicated an increase from the post-workshop survey in October 2018. A similar waning of the effect of antimicrobial stewardship over time has previously been reported. In a cluster randomized trial by Gerber et al. [7,8], an onsite education session followed by one year of personalized feedback reduced prescribing rates for broad-spectrum antibiotics in pediatric outpatients [7]. However, this reduction gradually disappeared and reverted back to baseline levels 18 months after discontinuation of the feedback [8]. Similarly, in other randomized trials by Meeker et al. [9] and Linder et al. [10], behavioral interventions reduced antibiotic treatment of adult viral acute respiratory tract infections [9], but this effect waned within 12 months of ending the studies [10].

Notably, in the present study, the reduction in the prescribing rate recovered to -45.6% in autumn 2019, which was concomitant with the second workshop, suggesting the effectiveness of the second workshop. These observations suggest the importance of repetitive and consecutive antimicrobial stewardships. However, the relative reduction rate recovered to -21.4% in winter 2019 (from January to March 2020). This observation indicated that the effect of the second workshop was smaller than that of the first workshop. However, regarding this observation, we need to consider the possible effect of the coronavirus disease 2019 pandemic. Antibiotics are not prescribed for viral infections, including adenovirus and coxsackievirus infections [11] in the summer and influenza infections [12] in the winter, which are seasonally observed in Japan [13,14]. Therefore, the antibiotic prescribing rate in emergency rooms was generally lower in the summer and winter, reflecting the seasonal outbreaks of viral illnesses observed in 2017 and 2018. However, in the 2019 winter season (January to March 2020), because of intensified prevention measures against the coronavirus disease 2019 pandemic, influenza activity declined in early February 2020 and remained at an extremely low level in March 2020. Therefore, the antibiotic prescribing rate in the 2019 winter season could have been much higher than that in previous winter seasons. Under these circumstances, the relative reduction rate in the 2019 winter season might have been underestimated.

In Japan, third-generation cephalosporins and macrolides account for the majority of oral antimicrobials administered to children [4,15]. In a nationwide study of outpatient oral antibiotic prescriptions using the national health claims database of 2013-2016 [4], third-generation cephalosporins and macrolides accounted for 36% and 38% of oral antimicrobials, respectively. In the present study, in the baseline year of 2017, cephems (mostly third-generation cephalosporins) and macrolides accounted for 53.8% and 18.2% of the total prescribed antibiotics in both centers, respectively. Therefore, a reduction in the prescribing of cephems is another major objective for antimicrobial stewardship in Yamanashi Prefecture. Notably, the prescribing rates of cephems dramatically decreased from 9.8% in 2017 to 5.7% (41.6% reduction) and 5.3% (46.1% reduction) in 2018 and 2019, respectively. As a result, cephems accounted for 46.4% and 43.4% of total antibiotic prescriptions in 2018 and 2019, respectively. Although the prescribing rate of cephems decreased during the study period in Yamanashi Prefecture, prescriptions still remained at a relatively high level, indicating the requirement of continuous antimicrobial stewardship.

The present study has several limitations. First, our study of antibiotic prescriptions in two emergency rooms was a simple historical comparison between the baseline year and the study years. Trends in activities of seasonal viral infections such as influenza could be similar but not identical in each year. Therefore, careful interpretation is required in this historical comparison. Second, we could not identify which activity was responsible for the reduction in prescribing antibiotics, although the prescription of oral antibiotics in the two emergency rooms was greatly reduced in the study years, consistent with the timing of the workshops. Moreover, baseline knowledge regarding antibiotic prescribing was not directly assessed for each pediatrician enrolled in this study. Third, we did not directly evaluate the effect of the workshop on antimicrobial prescriptions for each participant. The reduction in the antibiotic prescribing rate might be more significant in workshop participants than in nonparticipants. However, we could not evaluate individual prescriptions. Fourth, we were unable to evaluate the appropriateness of each prescription, although we found a reduction in the antibiotic prescribing rate, particularly the prescription of cephems, in the study years. Fifth, since the number of centers and pediatricians participating in this survey was limited, this study may underestimate the scope or true patterns of antibiotic prescribing across all emergency sites in the country. Sixth, we could not evaluate potential confounding by other preparatory local activities or by overlapping shifts and physicians working at both centers.

## Conclusions

Concomitant with regional stewardship efforts and national activities, this study observed a marked reduction in the prescribing rate of oral antibiotics in two pediatric emergency rooms from 2017 to 2018/2019. Although the antibiotic preferences (determined by questionnaire) of pediatricians considerably changed (to lower antibiotic prescribing rates) immediately after each workshop, the effect of the workshops gradually waned. These observations indicate the importance of continuous antimicrobial stewardship.

## Appendices

**Table 3:** Questionnaire

Do you prescribe antibiotics in the following cases? Please check either Yes or No.	
A) Afebrile toddler with nasopharyngitis (excluding streptococcal pharyngitis)	☐Yes ☐No
B) Three-day febrile toddler who complains of nasopharyngitis (excluding streptococcal pharyngitis)	☐Yes ☐No
C) Afebrile toddler who complains of wet cough with clear post-nasal discharge	☐Yes ☐No
D) Three-day febrile toddler who complains of wet cough without clear post-nasal discharge	☐Yes ☐No
E) Afebrile toddler who complains of wet cough with purulent post-nasal discharge	☐Yes ☐No
F) Three-day febrile toddler who complains of wet cough with purulent post-nasal discharge	☐Yes ☐No
G) Afebrile toddler who complains of mild otalgia with erythema in the eardrum	☐Yes ☐No
H) Afebrile school-aged child who complains of wet cough without wheezing	☐Yes ☐No
I) Three-day febrile school-aged child who complains of wet cough with wheezing	☐Yes ☐No
J) Afebrile female toddler who complains of increased urinary frequency and dysuria	☐Yes ☐No
K) Afebrile school-aged child who complains of abdominal pain and watery (non-bloody) diarrhea without vomiting	☐Yes ☐No
L) Three-day febrile school-aged child who complains of abdominal pain and watery (non-bloody) diarrhea without vomiting	☐Yes ☐No
M) Afebrile toddler who complains of multiple impetiginous lesions (impetigo)	☐Yes ☐No

## References

[1] Ohmagari N (2019). National Action Plan on Antimicrobial Resistance (AMR) 2016-2020 and relevant activities in Japan. Glob Health Med.

[2] Muraki Y, Yagi T, Tsuji Y (2016). Japanese antimicrobial consumption surveillance: first report on oral and parenteral antimicrobial consumption in Japan (2009-2013). J Glob Antimicrob Resist.

[3] Hsia Y, Sharland M, Jackson C, Wong ICK, Magrini N, Bielicki JA (2019). Consumption of oral antibiotic formulations for young children according to the WHO Access, Watch, Reserve (AWaRe) antibiotic groups: an analysis of sales data from 70 middle-income and high-income countries. Lancet Infect Dis.

[4] Kinoshita N, Morisaki N, Uda K, Kasai M, Horikoshi Y, Miyairi I (2019). Nationwide study of outpatient oral antimicrobial utilization patterns for children in Japan (2013-2016). J Infect Chemother.

[5] Okubo Y, Nishi A, Uda K (2023). Financial incentives for infection prevention and antimicrobial stewardship to reduce antibiotic use: Japan's nationwide observational study. J Hosp Infect.

[6] Iwamoto N, Morisaki N, Uda K, Kasai M, Kodama EN, Ohmagari N, Miyairi I (2022). Change in use of pediatric oral antibiotics in Japan, pre- and post-implementation of an antimicrobial resistance action plan. Pediatr Int.

[7] Gerber JS, Prasad PA, Fiks AG (2013). Effect of an outpatient antimicrobial stewardship intervention on broad-spectrum antibiotic prescribing by primary care pediatricians: a randomized trial. JAMA.

[8] Gerber JS, Prasad PA, Fiks AG, Localio AR, Bell LM, Keren R, Zaoutis TE (2014). Durability of benefits of an outpatient antimicrobial stewardship intervention after discontinuation of audit and feedback. JAMA.

[9] Meeker D, Linder JA, Fox CR (2016). Effect of behavioral interventions on inappropriate antibiotic prescribing among primary care practices: a randomized clinical trial. JAMA.

[10] Linder JA, Meeker D, Fox CR, Friedberg MW, Persell SD, Goldstein NJ, Doctor JN (2017). Effects of behavioral interventions on inappropriate antibiotic prescribing in primary care 12 months after stopping interventions. JAMA.

[11] Kuo KC, Yeh YC, Huang YH, Chen IL, Lee CH (2018). Understanding physician antibiotic prescribing behavior for children with enterovirus infection. PLoS One.

[12] Rao S, Lamb MM, Moss A (2021). Effect of rapid respiratory virus testing on antibiotic prescribing among children presenting to the emergency department with acute respiratory illness: a randomized clinical trial. JAMA Netw Open.

[13] Sakamoto H, Ishikane M, Ueda P (2020). Seasonal influenza activity during the SARS-CoV-2 outbreak in Japan. JAMA.

[14] Takahashi S, Metcalf CJ, Arima Y (2018). Epidemic dynamics, interactions and predictability of enteroviruses associated with hand, foot and mouth disease in Japan. J R Soc Interface.

[15] Uda K, Okubo Y, Kinoshita N, Morisaki N, Kasai M, Horikoshi Y, Miyairi I (2019). Nationwide survey of indications for oral antimicrobial prescription for pediatric patients from 2013 to 2016 in Japan. J Infect Chemother.

